# Systematic review on spheroids from adipose‐derived stem cells: Spontaneous or artefact state?

**DOI:** 10.1002/jcp.30892

**Published:** 2022-10-09

**Authors:** Anna Barbara Di Stefano, Valentina Urrata, Marco Trapani, Francesco Moschella, Adriana Cordova, Francesca Toia

**Affiliations:** ^1^ BIOPLAST‐Laboratory of BIOlogy and Regenerative Medicine‐PLASTic Surgery, Plastic and Reconstructive Surgery Unit, Department of Surgical, Oncological and Oral Sciences University of Palermo Palermo Italy; ^2^ Department of Surgical, Oncological and Oral Sciences, Unit of Plastic and Reconstructive Surgery University of Palermo Palermo Italy; ^3^ Department of D.A.I. Chirurgico, Plastic and Reconstructive Unit Azienda Ospedaliera Universitaria Policlinico “Paolo Giaccone” Palermo Italy

**Keywords:** 3D cultures, adipose stem cells, biomaterials, hanging drop, spheroids, spinner flask

## Abstract

Three‐dimensional (3D) cell cultures represent the spontaneous state of stem cells with specific gene and protein molecular expression that are more alike the in vivo condition. In vitro two‐dimensional (2D) cell adhesion cultures are still commonly employed for various cellular studies such as movement, proliferation and differentiation phenomena; this procedure is standardized and amply used in laboratories, however their representing the original tissue has recently been subject to questioning. Cell cultures in 2D require a support/substrate (flasks, multiwells, etc.) and use of fetal bovine serum as an adjuvant that stimulates adhesion that most likely leads to cellular aging. A 3D environment stimulates cells to grow in suspended aggregates that are defined as “spheroids.” In particular, adipose stem cells (ASCs) are traditionally observed in adhesion conditions, but a recent and vast literature offers many strategies that obtain 3D cell spheroids. These cells seem to possess a greater ability in maintaining their stemness and differentiate towards all mesenchymal lineages, as demonstrated in in vitro and in vivo studies compared to adhesion cultures. To date, standardized procedures that form ASC spheroids have not yet been established. This systematic review carries out an in‐depth analysis of the 76 articles produced over the past 10 years and discusses the similarities and differences in materials, techniques, and purposes to standardize the methods aimed at obtaining ASC spheroids as already described for 2D cultures.

## Graphical abstract

Our proposed model of spheroid isolation from liposuction fat. The tissue disintegration releases the pre‐existing spheroids as intact structures. This could be the ideal technique to culture cells in suspension because it avoids a preliminary adhesion isolation step and thus an upstream cell selection. In addition, spheroids can be differentiated.

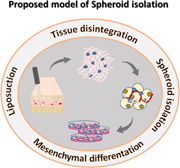

## 1 INTRODUCTION

### 1.1 Adipose stem cell (ASCs) spheroids

Two‐dimensional (2D) cell cultures are a very useful tool to perform in vitro studies in the field of oncology, stem cell biology and tissue engineering. Adhesion cultures are generally created by means of simple, standardized techniques requiring a support/substrate (flasks, multiwells etc.) and a specific media that employs fetal bovine serum as an adjuvant for cell adhesion and proliferation. Recently, researchers have asked if adhesion cultures manage to properly represent the original cells condition in in vivo tissues. This questioning has lead to a progressive replacement of 2D cultures in favor of three‐dimensional (3D) cell cultures with protein expression patterns and intercellular junctions that are more alike in vivo conditions. Today, these 3D cell cultures have become a very attractive system for the scientific community (Abbott, 2003), however experts do not all agree on these in vitro procedures. ASCs represent promising cell therapies for regenerative medicine and immunomodulation (Agrawal et al., 2014; Pappalardo et al., 2019). Laboratories throughout the world that are currently studying 3D cultures adopt a personal, nonstandardised technique albeit often achieving the same final evaluations, namely that 3D spheroids feature superior characteristics compared to 2D classical ASCs (A. Di Stefano, Montesano et al., 2020; A. B. Di Stefano et al., 2018; A. B. Di Stefano, Grisafi et al. 2020; A. B. Di Stefano et al., 2015; S. Kim, Han, et al., 2018).

Does the spheroid already exist in the tissue or is it the result of a later union or rearrangement of individual cells? This question continues to stand as specialist literature currently available is not conclusive.

This systematic review aims to summarize evidence from studies carried out in the past decade on ASC spheroids. This study reviews all the techniques used for their isolation and/or formation with specific plate, scaffold systems, or nonadhesion methods and discusses the benefits and drawbacks of the different techniques.

## 2 MATERIALS AND METHODS

A systematic literature search was conducted in the PubMed database for articles published from April 2011 to February 2021. The following key‐words were used: “spheroids [Title/ABSTRACT] AND adipose [Title/ABSTRACT] AND stem cells [Title/ABSTRACT],” species “HUMAN.” Selection of the articles was carried out based on the following inclusion and exclusion criteria:

(i) Iinclusion criteria: preclinical or clinical research papers concerning the potential regenerative role of S‐ASCs culture.
(ii) Exclusion criteria: reviews and papers that did not directly refer to human ASC spheroids studies.

Two reviewers independently screened all search results, abstracts, and full texts. Further searches included relevant references from selected articles. Data concerning the type of paper, cell isolation, culture methods, and results were extrapolated from the selected articles.

Data were analyzed to summarize current evidence on the following queries:

(i) What technical methods that have been developed in the past decade to create 3D cellular ASC aggregates/spheroids?
(ii) What are the benefits in terms of regenerative capacities of 3D‐ASCs compared to traditional 2D‐ASC cultures?
(iii) Why does not a standardized method of 3D‐ASC isolation and in vitro culture exist today?

## 3 RESULTS

### 3.1 Common culture methods using specific technical conditions (ultralow/low adhesion or concave multiwell)

Many authors isolate ASCs from liposuction, culture them in traditional adhesion conditions, trypsinize them and then take the cells to 3D conditions. Table 1 lists the selected studies using common culture methods including ultralow/low adhesion or concave multiwell to form ASC spheroids featuring different sizes and oxygen concentrations.

**Table 1:** Characteristics of selected studies using common culture methods ultralow/low adhesion or concave multiwall

Author	In vitro culture	Substrates/scaffolds	Results
Fennema et al. (2018)	Liposuction (thighs) I−II passages of 2D	1.5 × 10^6^ cells using agarose micro‐molds with basic medium	Study of osteoblastic differentiation	
Guo et al. (2015)	Liposuction (abdomen and thighs, female, mean age of 35 years old) N.A. passaged 2D (3.5 × 10^5^ cells)	Agarose micro‐molds with LG‐DMEM and well rubber of 400 µm diameter, called 3D Petri dishes of agarose. Silicone microwells. 81 wells rubber micro‐molds with diameter in 400 µm	Neural differentiation study	
Coyle et al. (2019)	Commercially VI−VII passage of 2D (0.5, 1.0, 2.5, and 5.0 × 10^6^ cells/ml)	Agarose hydrogel micro‐molds with 35 concave of 800 µm diameter and 800 µm depth	Cell ischemic model
De Moor et al. (2018)	Commercially III−VI passages of 2D (5000 cells/cm^2^)	High‐throughput non‐adhesive 3% w/v agarose microwell system in PDMS. One mold contained 2865 pores with a diameter of 200 μm and a depth of 220 μm each. 500 μl of cell suspension containing 7.5 × 10^5^ cells (262 cells per pore). For angiogenic study 1.0×10^6^ cells (350 cells per pore).	Angiogenic study in monoculture and coculture ASC/HUVEC/HFF	
Cho et al. (2017)	Commercially V passages of 2D (10^5^ cells)	PDMS‐based concave microwell, located into methylcellulose‐coated supports in 96‐well plates, with a diameter of 300 µm coated with 3% BSA. Each concave microwell contained 64 holes	Regeneration of lung tissues
No et al. (2012)	Liposuction 2D	PDMS‐based concave micromolds of 500 µm diameter coated with 3% BSA overnight.	3D coculture (ASC + hHEP) effect on hepatocyte function and spheroid formation	
G. H. et al. (2018)	N.A. passages of 2D (2 × 10^6^ cells)	A microwell array, concave microwells, 30 × 30 through‐hole array, several hundred micrometer‐order steel beads, and magnetic force to fabricate 900 microwells in a 3 cm × 3 cm PDMS	Spheroid formation
Rumiński et al. (2019); Rumiński et al. (2020)	Liposuction II−III passages of 2D (1 × 10⁶ cells/cm^₂^)	Pluronic F‐127 solution, 3D porous poly PS scaffolds	Osteogenic study	
Gimeno et al. (2017)	Liposuction (abdomen)	3.5 × 10^6^ cultured in nonadherent plastic diches	Study of immonomodulatory capacity
Winter et al. (2003)	Liposuction (total hip arthroplasty, mean age 55,3 years)	3–5 × 10^5^ cells seeded in culture flasks with a MSC culture medium	chondrocyte differentiation study	
Hong et al. (2020)	Commercially V passage 2D 5000 cells cm^–2^	bio‐functional matrix PS‐MBP‐FGF2: polystyrene (PS) surface with basic fibroblast growth factor (FGF2) bound to maltose binding protein (MBP).	Spheroids formation	d formation
Furuhata et al. (2016)	Commercially, III−V passages of 2D (3.2 × 10^4^ cells/cm^2^)	96‐well micropatterned culture plates	Study of cell aggregation effect	
Skiles et al. (2013); Skiles et al. (2015)	Commercially III−VI passages of 2D	5000 (5 k), 10,000 (10 k), 20,000 (20 k), or 60,000 (60 k) cells were pipetted into 0.5 ml, siliconized, screw‐cap microcentrifuge tubes and centrifuged at 500 rcf for 2 min. encapsulated within the PEG hydrogels	Spheroid size in normoxic (20%) and hypoxic (1% and 2%) condition in monoculture and HUVEC cocultures.
Oberringer et al. (2018)	Commercially V passages of 2D (10,000 cells/ml)	96‐well plates with cell‐repellent surface	Angiogenic study in monoculture and coculture with HDMEC	
Parshyna et al. (2017)	Commercially ASC about 500 cells per spheroid	Nonadherent round bottom 96‐well plates, embedded into a collagen matrix (Matrigel) and treated with omega‐3 fatty acids for 24 h.	omega‐3 fatty acids in angiogenic study of coculture with HUVEC

Abbreviations: ASC, Adipose derived stem cell; BSA, Bovine serum albumin; DMEM, Dulbecco's Modified Eagle's Medium;‎ HDMEC, Human Dermal Microvascular Endothelial Cells; HFF, Human foreskin fibroblasts; HUVEC, Human Umbilical Vein Endothelial Cells; LG‐DMEM, Low‐Glucose Dulbecco's Modified Eagle's Medium; PDMS: Polydimethylsiloxane;  PEG, Plyethylene glycol; PS, polystyrene.

Using agarose micromolds with basic medium, Fennema et al. (2018) generated ASC spheroids adding LG‐DMEM (Low‐Glucose Dulbecco's Modified Eagle's Medium) and a 400 µm diameter well rubber. Guo et al. instead formed 3D spheroids whose aggregates were then removed and transferred to the nonadhesive plastic Petri dishes called agarose 3D Petri dishes. This innovative method was used to overcome poor post thaw cells and improve the viability and neural differentiation potential of 3D‐ASCs (Guo et al., 2015). Likewise, Coyle et al. carried out an analysis on different spheroids sizes (115, 135, 175, and 215 υm radius) demonstrating, through in vitro culture and mathematical modeling, that the glucose availability and absence of oxygen improved the spheroids viability through anaerobic glycolysis (Coyle et al., 2019).

Using ultrapure agarose with PDMS (polydimethylsiloxane), De Moor et al. generated spheroids with 7.5 × 10^5^ cells, approximately 262 cells per pore and tested two different fusions: in suspension (in U‐shaped wells) and in matrigel. The aim was to evaluate the aggregation behavior in cocultures with ASCs, Human Umbilical Vein Endothelial Cells (HUVECs), and human foreskin fibroblasts in a 10‐day time span (De Moor et al., 2018). By means of a similar system, Cho et al. generated spheroids into methylcellulose‐coated supports in 96‐well plates featuring a diameter of 300 µm, further coated with bovine serum albumin (BSA) aiming to prevent cell attachment (Cho et al., 2017). No and collaborators instead coated the plates with 3% BSA obtaining spheroids with a different diameter (500 μm), fabricating them by employing the soft lithography technique. They also discovered that hepatocyte spheroids acquired an improved liver‐specific function when cocultured with hASC spheroids (No et al., 2012). Finally, Lee et al. used concave microwells with PDMS on aluminium plates acting as a mold, placing them into 24‐well plates and generating ASCs after 24 h (G. H. Lee et al., 2018).

Rumiński et al. applied a scaffold free, low‐adhesion technique to generate spheroids by using plates treated with Pluronic F‐127 aqueous solution, but also by employing a common 3D porous polystyrene (PS) scaffold as an additional method. They compared the osteogenic capacity of the two different 3D‐ASC models with 2D cultures showing that the ASC proliferation increased in PS scaffolds similar to the control 2D condition, but the osteogenic marker expression was observed in scaffold‐free ASC spheroids. Nonetheless, mineral production was higher in 3D PS and in control 2D cultures as the mineralization process required a substrate (Rumiński et al., 2019). Consequently, the effect of a cyclic adenosine monophosphate (cAMP) pathway activity on the osteoblastic differentiation of ASC spheroids compared to 2D cultures was also subject to observation. Early osteogenesis was inhibited when cAMP was upregulated. Moreover, the protein kinase A activity may have a role in addressing ASCs to the osteogenic differentiation (Rumiński et al., 2020).

In nonadherent plastic dishes, Gimeno et al. obtained Muse‐AT cell spheroids, a pluripotent stem cell subpopulation of adipose derived stem cells (ADSCs), and studied their spontaneous clustering until they reached a diameter between 50 and 150 μm after 72 h in culture (Gimeno et al., 2017). Similarly, Winter et al. (2003) generated spheroids through a spontaneous aggregation of cells plated at a density of 3–5 × 10^5^ cells on a 48‐well plate after 1−2 days.

In a biofunctional matrix of PS coated with basic fibroblast growth factor (FGF2) and maltose binding protein, Hong et al. generated functional 3D hepatocytes with hASCs as supporting cells that were cocultured with mouse induced hepatic precursor cells (miHeps) (Hong et al., 2020).

Small spheroids with average spherical shapes (100 μm diameter) were obtained when a seeding density of 100.000 cells/cm was reached in 96‐well plates containing circular adhesive domains. In this study, the 3D condition of the ASCs was associated with an increase of the VEGF‐A and IL‐8 expressions as regards to wound healing (Furuhata et al., 2016). The capacity to form spheroids in response to different oxygen concentrations is a consolidated fact. Culture geometry, depending on spheroid size, is essential to hypoxia effects; each spheroid counted a different number of cells and was placed at different oxygen concentrations to observe the VEGF expression. An increased VEGF secretion was observed in the spheroids in 2% oxygen conditions compared to a 20% oxygen culture condition (Skiles et al., 2013), (Skiles et al., 2015). Oberringer et al. studied the cocultures of human dermal microvascular endothelial cells (HDMEC) and ASC spheroids using pretreated repellent‐surface wells during tissue healing. Tissue reoxygenation appears subject to the strong paracrine role of ASCs and on the 3D cell−cell interaction with HDMECs (Oberringer et al., 2018). Due to the same angiogenic capacity of the 3D‐ASCs, Parshyna et al. studied the role of several omega‐3 fatty acids. In particular, docosahexaenoic acid promoted the sprouting activity of ASCs supporting the formation of microvascular networks in cocultures with HUVECs into a collagen matrix (Parshyna et al., 2017).

### 3.2 Common culture methods of ASC spheroids (hanging drop or spinner flask)

Table 2 summarizes the conventional techniques used to form ASC spheroids: hanging drop or spinner flask. A widespread technique is the hanging drop method that is based on the natural predisposition of cells to form 3D aggregates needless of scaffolding. A drop is formed in an inverted plate and held in place due to surface tension. Similar to this case, there are many studies that adopt this technique prooviding for some modifications: spheroid formation times, different drop volumes and abilities to differentiate towards mesenchymal lineages. Kapur et al. demonstrated a homogeneous distribution of the cells inside the spheroids. After 24 h from the drop insertion placed upside down, aggregates were formed and then maintained into a 24‐well ultralow attachment plate. Subsequently, the authors showed the upregulation of specific mesenchymal markers (RUNX2, AP, Collagen 1,2, aggregan, LPL, PPARG2, and FABP4) in the spheroids grown in osteoblastic, chondrocytic and adipogenic media compared to the control group (Kapur et al., 2012). Similarly, the Shen group formed multicellular aggregates from forty‐microliter droplets containing 50,000 ASCs and plating them in 96‐ultralow‐adhesion multiwell plates for 2 weeks. The result was a high spheroid capacity of osteoblastic differentiation with deposition of bone matrix in both in vitro and in vivo studies (F. H. Shen et al., 2013). Hutton and colleagues plated the adherent ASCs in 10 μl drops on inverted Petri dishes. After one night, the formation of spheroids was observed and were subsequently seeded on a fibrin gel with osteogenic and vascular media. It was found that spheroidal aggregates presented a greater number of vascular networks than the counterpart in 2D condition (Hutton et al., 2013). Xu et al. used drops with 25,000 cells and a volume of 35 microliters, plating them upside down for 3 days. The generated spheroids featured a greater potential for vascular regeneration in acute kidney damage (Xu et al., 2016). Another study on neurogenic erectile dysfunction evaluated spheroid formation after 5 days from the generation of the drops with 25 μl in volume (Xu et al., 2020).

**Table 2:** Characteristics of selected studies using common culture methods Hanging drop or Spinner flask for spheroids of adipose stem cells

Author	In vitro culture	Methods	Results
Kapur et al. (2012)	Liposuction (abdomen, average age 42.4 years, BMI of 30.14) V passages of 2D (2000 cells/cm^2^)	Hanging drop: 10, 20, 30, and 50 k cells in 24−48 h, then they transferred to ultralow 24‐well plates.	Stemness and differentiation study
F. H. Shen et al. (2013)	Liposuction III Passages of 2D (5 × 10^4^ cells/cm^2^)	Hanging drop: 50 k cells in 40ul of drop for 24 h, then they transferred to Ultralow 96‐well plates.	Osteogenic study
Hutton et al. (2013)	Liposuction (female, average age of 46 years, average BM1 of 29.1) II passages of 2D (N.A. cell/cm^2^)	Hanging drop: 400 k cells/ml spheroid in 10 μl drop fibrin gel	Vascularized bone tissue formation
Xu et al. (2016)	Liposuction 90% confluence of 2D (N.A. cell/cm^2^)	Hanging drop: 25 k cells in 35 μl drop	Vascular regeneration in acute kidney damage
Xu et al. (2020)	Liposuction	Hanging drop: 25 μl were used for 5 days to observe spheroid formation. on the third day, 1000 cells were recovered	Neurogenic erectile dysfunction studies
Berg et al. (2015)	Liposuction (female, age between 28−32 years old) IV passages of 2D (1.6 × 10^5^ cells/cm^2^)	Hanging drop: 2000 cells in 50 μl drop	Hippocampal neurogenesis and nigral neurodegeneration
Yoon et al. (2012)	Liposuction IV passages of 2D	Siliconized Spinner flask: 1.0 × 10^6^ cells/ml in 100 ml volume stirred at 40 rpm for 3 days	Cartilage formation
Williams et al. (2013)	Liposuction (abdominal) N.A. passages of 2D	Siliconized Spinner flask: 3.0 × 10^6^ cells/ml in 1,5% alginate at 5 rpm for 2 days and then increased to 10 rpm for 16 days.	Spheroid fabrication
Bhang et al. (2011)	Liposuction V passages of 2D (N.A. cell/cm^2^)	Siliconized spinner flasks: 6.0 × 10^5^ cells/ml at 70 rpm for 3 days.	Angiogenic study
Hoefner et al. (2020)	Commercially IV−V passage 2D (25,000 cells/cm^2^)	Orbital shaker at 50 rpm: 5000 cells/well in 96‐well plates coated with 1.5% agarose. Multicellular 3D spheroids obtained through the liquid overlay technique.	Adipogenic study

Berg et al. obtained spheroids in a suspended drop after 4 days of culture and compared them with monolayer cells to study their neural involvement in a Parkinson disease rat model injured by a 6‐hydroxydopamine (OHDA) injection. After 8 days, the transplantation of the monolayer ASCs in the substantia nigra of rats showed a significant increase in tyrosine hydroxylase, in the brain‐derived neurotrophic factor and in glial fibrillary acid protein levels, while the grafting of spheroids led to an increase in local microgliosis. Consequently, the monolayer culture cells induced a motor function improvement, while the spheroids caused local inflammation and suppression of hippocampal neurogenesis (Berg et al., 2015).

A different strategy for forming spheroids is the spinner flask whereas the fluid is put into turbulence, preventing adhesion and promoting cellular aggregation. Yoon et al. used this method to demonstrate the greater potential of 3D‐ASCs cultures compared to the 2D‐ASC cultures. Furthermore, they found an improved chondrogenic capacity of hASC spheroids placed in specific differentiation medium added with TGF‐b3, confirmed by the greater expression of SOX‐9, aggrecan and type II collagen compared to the monolayer culture (Yoon et al., 2012). Williams et al. bioprinted alginate spheroids of ASCs and transferred them into a spinner flask for 2 days. They found the absence of a necrotic core rather than homogenous dissemination of the bioprinted cells (Williams et al., 2013). Bangh et al. placed spheroids in hypoxic culture conditions with 1% oxygen for 3 days. In this condition, spheroids grew faster than monolayer culture and, focusing the attention on the treatment of the ischemic disease, the authors found increased expression levels of survival factors (CXCL12 and HIF‐1a) in response to the size of the spheroids. Indeed, apoptosis seems to be involved with the hypoxia phenomenon. The expression of the antiapoptotic factor, Bcl‐xL, in the spheroidal culture condition led to an increase of the adhesion molecules such as ICAM‐1, VCAM‐1, and PECAM‐1. These processes enhanced the angiogenic efficacy of stem cells therapy (Bhang et al., 2011).

Hoefner et al. investigated the adipogenic differentiation and the development of ECM in ASC spheroids comparing them to 2D cultures. They were cultured in growth cell medium during shaking at 50 rpm. After only 2 days of induction for the adipogenic lineages, they demonstrated an improved differentiation capacity of the ASC spheroids located in their own ECM (laminin and Col IV) compared to 2D cultures (Hoefner et al., 2020).

### 3.3 Novel culture methods to form spheroids of ASCs

Table 3 summarizes selected studies that used novel culture methods to form ASC spheroids.

**Table 3:** Characteristics of selected studies using novel culture methods to form spheroids of adipose stem cells

Author	In vitro culture	Substrates/scaffolds	Results
Feng et al. (2017)	Liposuction (abdomen or thighs, female, aged 25−27) 1 week of 2D (N.A. cell/cm^2^)	3%, 4%, and 5% HA gel in a syringe with (2 × 10^6^, 4 × 10^6^ and 6 × 10^6^) cells and medium	Stemness study
Hu et al. (2020)	Liposuction (abdomen or thighs) for up to IX passage of 2D	Non cross‐linked hyaluronic acid (HA) gel	Angiogenic study
Huang et al. (2011); Hsu et al. (2014); Cheng, Wang, et al. (2012); Cheng, Wang, et al. (2012); Chen et al. (2018)	Adipose tissue (abdomen, female, age 30–57, BMI 24.8) III−XIII passages of 2D culture (N.A. cell/cm^2^) (1 × 10⁴ cells/cm^2^)	Chitosan modified with HA, PVA, gelatin or co‐adiuvated with FGF‐2	Stemness study
Lin et al. (2020)	Commercially IV passage 2D	Porous 3D hybrid scaffold of chitosan and native cartilage ECM	Chondrogenic study
Ziane et al. (2012)	Adipose tissue (age 20−80) VI passage 2D (40.000 cells/cm^2^)	GNF thermosensitive Hydrogel 1.5 million cells/ml of gel	Osteogenic study
G. Kim, Jung et al. (2020)	Commercially V passage 2D	A thermoresponsive poly(N‐isopropylacrylamide) (PNIPAAm) hydrogel substrate (PHS) functionalized with poly (ethylene glycol) (PEG) hydrogel	Spheroid formation
Seo et al. (2014)	1.25, 2.5, and 5.0 × 10^5^ cells/ml of 3D	Water adhesive surface of the Pd/Si NWs gas‐sensitive Pd, Si, NWs coated DTS	Stemness and angiogenic studies
Labriola et al. (2018)	Liposuction (abdomen and thigh, female, 56 year old) III passages of 2D (N.A. cell/cm^2^)	CMMPs formed to developed PAAm	Adipogenic study
J. Lee et al. (2020)	Commercially IV passage 2D	Bio‐mineral coated fibers loaded of platelet derived growth factor (PDGF) molecules	Osteogenic and angiogenic studies
K. Zhang et al. (2017)	Liposuction (abdomen, mean age 30 years) III passages of 2D (1 × 10⁶ to 5 × 10⁷ cells/ml)	PLGA	Angiogenic study
J. Wu et al. (2017)	Liposuction (abdomen, mean age 30 years) III passage 2D 5 × 10⁵ cells/ml	PGA cross‐linked with OEGs	Angiogenic study
Ahmad et al., 2018	VII passage of 2D	4% PLLA fibers electrospun in a DCM and TFE blend	bone tissue engineering studies
Ma et al. (2014)	Adipose tissue (abdomen) III passages of 2D (N.A. cell/cm^2^)	Collagen (3 mg/ml) and OPF (3400 g/mol)	Osteogenic study
Newman et al. (2020)	Liposuction	collagen and elastin‐like polypeptide (ELP) scaffolds	Adipogenic study
Gurumurthy et al. (2017)	Liposuction (female) 80% confluence of 2D (N.A. cell/cm^2^)	Polymer ELP conjugated to PEI coating of TCPS 50,000 cells	Osteogenic study
Fitzgerald et al. (2020)	Adipose tissue (female)	ELP‐PEI method (two molecular weights: 800 and 25,000 g/mol) 26,000 cells/cm^2^	Adipogenic study
Chuang et al. (2020)	Adipose tissue 2D (20,000 cells/well)	(AuNR)‐PEG‐PEI (APP)/chlorin e6 (Ce6) system	Cancer therapy
Oliveira et al. (2018)	Commercially 2D (7000 cells/cm^2^)	Matrigel	Cancer therapy
Liu et al. (2019)	Commercially (200,000 cells m/L)	PIC hydrogel conjugated with the adhesive peptide GRGDS	Spheroid formation
Kundu et al. (2019) ^(59)^	Liposuction II passages 2D	3D hydrogel created from gellan gum (GG)‐silk fibroin	Tumor stroma study in osteosarcoma model
S. Kim, Park, Kim, et al. (2019); S. Kim, Park, Byun, et al. (2019)	Commercially II‐V passages 2D (0.5, 1, 2, and 4×10⁵ cells/cmᶟ)	all‐in‐one platform with HES or LoSH with Tet‐TA polymers coniugated with fibronectin in a PDMS master mold	Spheroid formation
S. Kim, Han et al. (2018)	70% confluence of 2D (N.A. cell/cm^2^)	StemFit 3D®: GMP 100 and 200 MPs/well, 1000 and 2000 cells/well	Spheroid formation
Ko et al. (2020)	Liposuction 5 × 10^5^, 1 × 10^6^, 1.5 × 10^6^ cells/ml	StemFit 3D®: 200, 600 and 400 µm well diameter.	Osteoarthritis study
Y. Wu et al. (2018)	Adipose tissue N.A. Passages of 2D (N.A. cell/cm^2^)	Microfabricated pTS 4% tubular alginate Porogens in 4% CaCl2. The cell/porogen mixture was 5:3 ratio.	Self‐assembly, chondrogenic and osteogenic studies
G. Kim, Jung et al. (2020)	Commercially (1.2 × 10^6^ cells/cm^3^)	Loading spheroids onto acellular, lyophilized and sterile human dermis thin sectioned acellular dermal matrix (tsADM). 8 mm diameter round tsADM was fixed at the center of the coverslip.	Spheroid formation
Silva et al. (2016)	Liposuction 2.5 × 10^5^ and 1 × 10^6^ of 3D cells	Lockyballs: synthetic micro‐scaffolds with porous wall, produced by two‐photon polymerization of Zr‐based hybrid‐photopolymer. Micro‐molded (2% agarose) hydrogel.	Spheroid formation
Iwazawa et al. (2018)	Commercially N.A. passage 2D (3 × 10^5^ cells/ml)	CellSaic: cell‐ and ‐scaffold‐forming mosaic scaffold with a bioabsorbable biomaterial RCP in EZ SPHERE, a micro‐fabricated plastic vessel containing micro‐ wells	Cell therapy for colitis
Shima and Makino (2020)	Commercially V‐VI passages 2D 5000 cells cm^–2^	A plate with multicavity structures and bland adhesive properties due to a fluorinated polyimide film (MicoCell)	Spheroid formation Angiogenic study
J. Park et al. (2017)	Commercially 5 × 10^5^ cells of 3D	CMS method: 100 microwells one spheroid per microwell. Internal surface is coated with pluronic copolymer solution. The top and bottom layers with PDMS. Initially 3000 RPM for 3 min rotation then 1000–2000 RPM for three days.	Spheroid formation
S. Zhang et al. (2015)	Adipose tissue 10^6^ cells/ml of 3D	A microgravity bioreactor at 25 rpm with ASCs until 5 days	Spheroid formation
Ayan et al. (2020)	Adipose tissues 1 × 10^6^ cells/ml	Aspiration‐assisted bioprinting (AAB) technique	Osteochondral study
I. S. Park et al. (2015); I. S. Park et al. (2016)	Commercially V−VIII passage of 2D (7.5 × 10^4^ cells/cm^2^)	LLLI at 660 nm wavelength 10 min daily from Day 1 to 14	Angiogenic study
K. Shen et al. (2020)	IV passage of 2D (5000 cells per well)	liquid overlay, which involves coating the surface of the culture dish with a layer of agarose.	Studies of the adipose microenvironment surrounding the kidney
Al‐Ghadban et al. (2020)	Liposuction (thighs) III passage 2D	Orbital shaker at 50 rpm: 30 × 10^3^ cells/cm^2^ in 12‐well plates coated with 1.5% Ultrapure Agarose solution	Studies on adipogenic potential, differentiation, and ECM remodeling
Hackenberg et al. (2013)	Liposuction 2D (2.000 cells/cm^2^)	nanoparticles of titanium dioxide and zinc oxide	Nanoparticle cytotoxicity study on hMSCs

Abbreviations: AuNR, old nanorods; Ce6, chlorin e6; CMMPs, Cell mimicking microparticles; CMS, Centrifugal microfluidic‐based spheroid; DCM, dichloromethane; DTS, Dodecylalkyltrichlorosilane; ELP, Elastin‐like polypeptide; G‐FF, Gelatin fragmented fibers; GMP, Gelatin neutral microparticles; GNF, Glycosyl‐nucleosyl‐fluorinated; GRGDS, Gly‐Arg‐Gly‐Asp‐Ser; HA, Hyaluronic Acid; HES, embossed surface; OEGs, Oligo (ethylene glycol)s; OPF, Oigo (poly(ethylene glycol) fumarate;  PAAm, Polyacrylamide; PDMS, Polydimethylsiloxane; PEI, Polyethylenimine; Pd, Palladium; PDMS, Poly (dimethylsiloxane); PEG, poly ethylene glicol; PIC, Polyisocyanides; PLGA, Poly (L‐glutamic acid); PLLA, Poly‐lactic Acid fibers; pTS, Porous tissue strands; LLLI, Low‐level light irradiation; LoSH: lotus seedpod‐inspired; NWs, Nanowires; RCP, Recombinant protein; Si, Silicon; TCPS, Tissue culture polystyrene; Tet‐TA, tyramine‐conjugated Tetronic; TFE, trifluoroethanol.

Several studies tested different hydrogels to form ASC spheroids by employing state of the art materials in the field of tissue engineering such as hyaluronic acid (Feng et al., 2017; Hu et al., 2020) and chitosan (Chen et al., 2018; Cheng, Chang, et al., 2012; Cheng, Wang, et al., 2012; Hsu et al., 2014; Huang et al., 2011; Lin et al., 2020), demonstrating an improved stemness gene expression compared to the traditional cultures in adhesion plates. The combination of these materials, or with other materials (FGF‐2 and PVA), was able to form 3D spheroids in a short period of time and showed better properties in stemness maintenance and an increased ability in stimulating angiogenic (Hsu et al., 2014; Hu et al., 2020) and chondrogenic (Huang et al., 2011; Lin et al., 2020) differentiations. Many materials were thermoresponsive such as GNF (glycosyl‐nucleosyl‐fluorinated) hydrogel (Ziane et al., 2012) or poly‐N‐isopropylacrylamide (PNIPAAm) (G. Kim, Jung et al., 2020). These allowed an ASC differentiation, in osteogenic terms, also in the absence of differentiating or water‐repellent materials (Pd/Si NWs) (Seo et al., 2014), capable of forming spheroids with a homogeneous size. The chemical and physical properties of the 3D‐composite (polyacrylamide [PAAm] with cell mimicking microparticles [CMMPs]) were studied during the adipogenic differentiation of 3D spheroids. The microparticles were distributed into the center of the spheroids for 24−48 h (Labriola et al., 2018).

Fibers of poly‐lactic acid (PLLA) (J. Lee et al., 2020) or Poly (l‐glutamic acid) (PLGA) (K. Zhang et al., 2017), alone or cross‐linked with OEGs (J. Wu et al., 2017), were created with a significant swollen hydrophilic network that weakens cell‐scaffold adhesion, leading ASCs to form spheroids. Ahmad et al. investigated fragmented PLLA fibers coated with gelatin fragmented fibers (Ahmad et al., 2018) or adenosine polydopamine intended for bone tissue engineering applications. Moreover, J. Lee et al. (2020) generated 3D spheroids with epigallocatechin gallate (EGCG)‐coated PLLA fibers and exposed them to H_2_O_2_ for 12 h proving that only in EGCG coated spheroids, cell viability and antioxidative enzyme expression were improved.

In the same manner, collagen with oligo(poly(ethylene glycol) fumarate) (OPF) (Ma et al., 2014) or elastin‐like polypeptide (ELP) (Newman et al., 2020) was used. The first study demonstrated that ASCs featured higher proliferation and osteogenic properties compared to BM‐MSCs into the scaffold when using the dispersed and the spheroid methods (Ma et al., 2014), while with ELP the hASCs differentiation towards the adipogenic lineage is strictly related to the spheroids morphology and scaffold properties (composition, density, and mechanical characteristics) (Newman et al., 2020).

Another study that used polypeptide similar to elastin (ELP), albeit conjugated to polyethylenimine (PEI), demonstrated that the spheroids grew on this support with a differentiation potential toward the osteogenic lineage that was greater compared to 2D‐ASC cultures (Gurumurthy et al., 2017). Fitzgerald et al. compared the ELP‐PEI method to suspension and ultra‐low attachment static cultures evaluating the generation of spheroids and their adipogenic differentiation. A significant reduction of spheroids was observed in both suspension and ultra‐low attachment static cultures due to the fusion of spheroids (Fitzgerald et al., 2020). A new strategy for tumor therapy used gold nanorod (AuNR) (photothermal agent)‐PEG‐PEI (APP) and Chlorin e6 (Ce6) (photodynamic agent)—loaded ADSCs. After irradiation of the APP/Ce6 agents (808 and 660 nm) allowing activation, the role of ASCs was demonstrated in tumor migration, tropism and anticancer properties in in vitro and in vivo models (Chuang et al., 2020).

Oliveira et al. obtained spheroids using Matrigel, a typical scaffold in tumor studies. They examined the involvement of kinin receptors 1 (B1R) in direct cross‐talk between two distinct glioblastoma phenotype cells (U87 and U373 cells) and MSCs having two different origins (BM‐MSCs and ASCs). The results showed that glioblastoma cells increased their proliferation rate when mixed with BM‐MSC spheroids, but not when in contact with ASC ones (Oliveira et al., 2018). A further class of synthetic biomaterials used for spheroid formation is the PIC (polymeric hydrogels of polyisocyanides). By encapsulating several cellular lineages in PIC gel conjugated with the adhesive peptide Gly‐Arg‐Gly‐Asp‐Ser, and also varying length and concentration, Liu et al. observed that smooth muscle and tumor cells generated spheroids that proliferated in relation to the polymer density, while ASCs did not form spheroids but showed an elongated morphology, thus concluding that different cell behaviors can occur due to the extracellular matrix expression, organization and interaction with the hydrogel (Liu et al., 2019). Kundu et al. generated spheroids using a construct made with gellan gum (GG)‐silk fibroin (S) hydrogels seeding both ASCs and cancer cells on the top to investigate if ASCs were capable of inducing cancer cells to form spheroids. Authors found cancer cell spheroids inside the core of the compartmentalized hydrogel system (Kundu et al., 2019).

Based on mechanical structure or new systems, several innovative techniques were developed to form spheroids of ASC characterized by controlled size, higher stemness, and improved differentiation abilities, such as the following:

‐ a method defined “all‐in‐one platform” with hydrogels with an embossed surface (HES) or lotus seedpod‐inspired (LoSH) (S. J. Kim, Park, Byun, et al., 2019; S. J. Kim, Park, Kim, et al., 2019);
‐ StemFit 3D, based on a 389‐microwell plate employed with gelatin microparticles (GMP) (Y. Kim, Baipaywad et al., 2018; Ko et al., 2020);
‐ microfabricated porous tissue strands (pTS) (Y. Wu et al., 2018);
‐ human dermis named thin sectioned acellular dermal matrix (tsADM) (J. H. Kim & Lee, 2020),
‐ lockyballs: microscaffolds with a porous wall and interlockable hooks produced by using two‐photon polymerization (Silva et al., 2016);
‐ CellSaic platform, based on a biodegradable material, a recombinant Recombinant protein (Iwazawa et al., 2018);
‐ MicoCell, a plate with bland adhesive properties, due to a fluorinated polyimide film (Shima & Makino, 2020).

As opposed to the use of scaffolds, some studies developed novel scaffold‐free methods to obtain spheroids:

‐ a centrifugal microfluidic‐based spheroid formation method composed of a rotating platform for a dynamic culture (J. Park et al., 2017);
‐ a microgravity bioreactor (S. Zhang et al., 2015);
‐ an aspiration‐assisted bioprinting technique (Ayan et al., 2020);
‐ LLLI (low‐level light irradiation) in low adherence PS plates method (I. S. Park et al., 2015, 2016).

Another scaffold‐free technique is the liquid overlay thanks to which spheroids can be generated through the use of nonadhesive plastic surfaces or adhesive surfaces functionalized with hydrophilic nonadhesive polymers such as agar or agarose.

By applying this technique for the first time, Shen et al. generated cocultures of adipocyte spheroids both with M0 macrofages to investigate their capability in invading the adipose tissue in vitro (K. Shen et al., 2020). Al‐Ghadban et al. instead obtained ASC spheroids from both healthy and lipedema patient seeded at a density of 30 × 10^3^ cells/cm^2^ in 12‐well plates coated with Ultrapure Agarose, and plating them on an orbital shaker at 50 rpm (Al‐Ghadban et al., 2020). Coating a 96‐multiwell plate with 0.1% soft agar and adding 6 × 10^3^ cells to each well, Hackenberg et al. generated hMSC spheroids (Hackenberg et al., 2013).

## 4 DISCUSSION

Spheroid is an element with its own defined and unique characteristics regardless of the method used for its formation. The size of the spheroid is critical because, increasing in size, the levels of expression of hypoxic factors stimulating angiogenesis and of antiapoptotic genes increase as well. Therefore, spheroids contrast the anoikis phenomena better than monolayer cultures. Another important factor to consider in in vitro cultures is that during the detachment process, the trypsin action on the single‐layer of cells causes a great loss of laminin, fibronectin and structural components of the extracellular matrix (Bhang et al., 2011). Nowadays, these 3D cell cultures have become a very attractive system for the scientific community (Abbott, 2003). Indeed, in the field of regenerative medicine, adherent cell cultures of ASCs are still traditionally used both in in vitro and in vivo studies. These cells were largely characterized and this was demonstrated by the fact that many factors can influence their properties. Amongst these factors, there are the characteristics of native adipose tissue (visceral or subcutaneous), of site donors (breast, thigh, abdomen and hip), patient characteristics (age, body mass index [BMI], and sex) as well as the ASC isolation and culture conditions (media, supplemented factors, and scaffolds) (Prieto González, 2019). These factors have not still been analyzed on spheroids yet, however they were subject to an in‐depth examination in the 76 papers analyzed. In most of the articles, subcutaneous fat from the abdomen and thighs was used. Some did not describe the fat site donor, while others did not use fat from patients but employing commercial cell cultures. A single work used fat from the hip after arthroplasty (Winter et al., 2003). Moreover, very few authors detailed the BMI, whilst cell yields do not seem to be correlated with age and gender (only women). For the first time, we correlated the factors mentioned above with the yield of spheroids from ASCs, which we called SASCs. The cell yields of nine‐two subcutaneous fat samples from abdomen, flanks, thighs and breast were statistically analyzed, also correlating age, sex, BMI, and the fat harvesting method. Male patients and thighs were the only two factors that affected the SASC yield (A. C. Di Stefano et al., 2022).

A fundamental question arises in this review; is it the spheroid that already exists in the tissue (spheroid isolation) or is it the result of a subsequent union or reorganization of individual cells (spheroid formation)? Regardless of the isolation/formation methods, the studies discussed here definitively established the better skills and potential of ASC spheroids when compared to the adherent cells in stemness and mesenchymal differentiation conditions. Results are controversial in the tumor microenvironment because ASCs seem to participate in tumor aggressiveness in osteosarcoma, but could also be a potential vehicle for antitumor therapies. We summarized different methods to generate 3D spheroids which are grouped in classic, canonical and innovative techniques. This proves that there is no standardized method for the isolation and in vitro culture of 3D‐ASCs.

In the last 10 years, scientific literature has established that 3D spheroids properly represent the original cells condition in in vivo tissues (Abbott, 2003). This would lead us to assume that spheroids already exist in the tissue as a 3D structure and can therefore be directly isolated, most likely through tissue disintegration. We believe that the ideal technique would be to culture cells in suspension without preliminary adhesion isolation or additional processes. In fact, this would lead to an upstream selection of cells that could form different spheroids. Among the classic techniques, only three studies (A. B. Di Stefano et al., 2015; Gimeno et al., 2017; Winter et al., 2003) and, amongst the canonical techniques, the hanging drop and spinner flasks, all obtain spheroids without requiring the first in vitro selection step under adherence conditions.

In the section concerning these innovative methods, we included multiple strategies for spheroid formation; from those featuring a physical nature such as LLLI, a bio‐chemical‐physical nature (PAAm with CMMPs), to scaffolds such as GNF hydrogel, PLLA‐G/mff, PLGA‐ADH hydrogel, PLGA‐OEGs, HA and HA‐chitosan, ELP‐PEI, and Pd/Si NWs systems. Furthermore, there is the engineering‐structural nature such as Bioreactors, Lockyballs, CellSaic, StemFit 3D, and pTS. Among these studies, only two involve the immediate use of 3D without passing through the adherence conditions such as the centrifugal microfluidic disk formulated to generate finely‐controlled multicellular spheroids (Park et al. 2017) and the Pd/Si NWs system performed with 3D‐ASCs obtained from the hanging drop method (Seo et al., 2014).

We believe that standardizing a single 3D‐ASC isolation technique, such as the spontaneous formation in ultra‐low culturing conditions without the need of special instruments, would be more reliable and reproducible solution.

Although we propose an ideal spheroid isolation technique, it is crucial to evaluate two others important aspects in this discussion: one highlights the importance of these techniques for the in vitro characterization of spheroids, however, on the other hand, it is important to find innovative techniques that involve the least manipulation and the highest yield of cells for prompt use in the clinical field.

**Figure 1:**
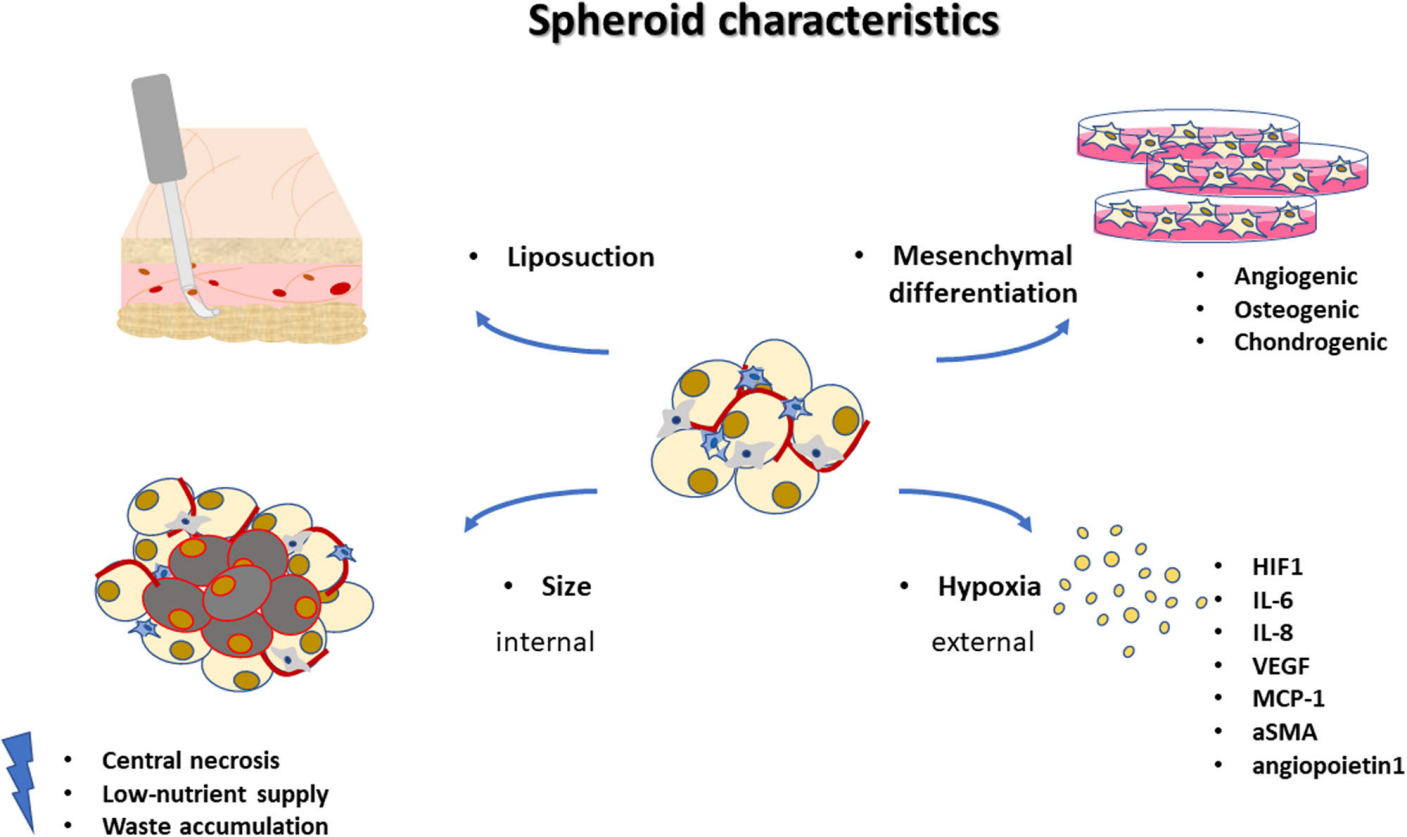
Summary of characteristics to form spheroids of adipose stem cells. Spheroids of adipose stem cells can derive from liposuction fat and can be differentiated in several mesenchymal lineages such as angiogenic, chondrogenic and osteogenic ones. Size and hypoxia effects are the major features concerning the formation and abilities of spheroids. Size mainly plays internal effects such as: central necrosis, low‐nutrient supply and waste accumulation whilst hypoxia has external ones such as the autocrine and/or paracrine factors production of HIF1, IL‐6, IL‐8, VEGF, MCP‐1, aSMA, and angiopoietin1 factors.

**Figure 2:**
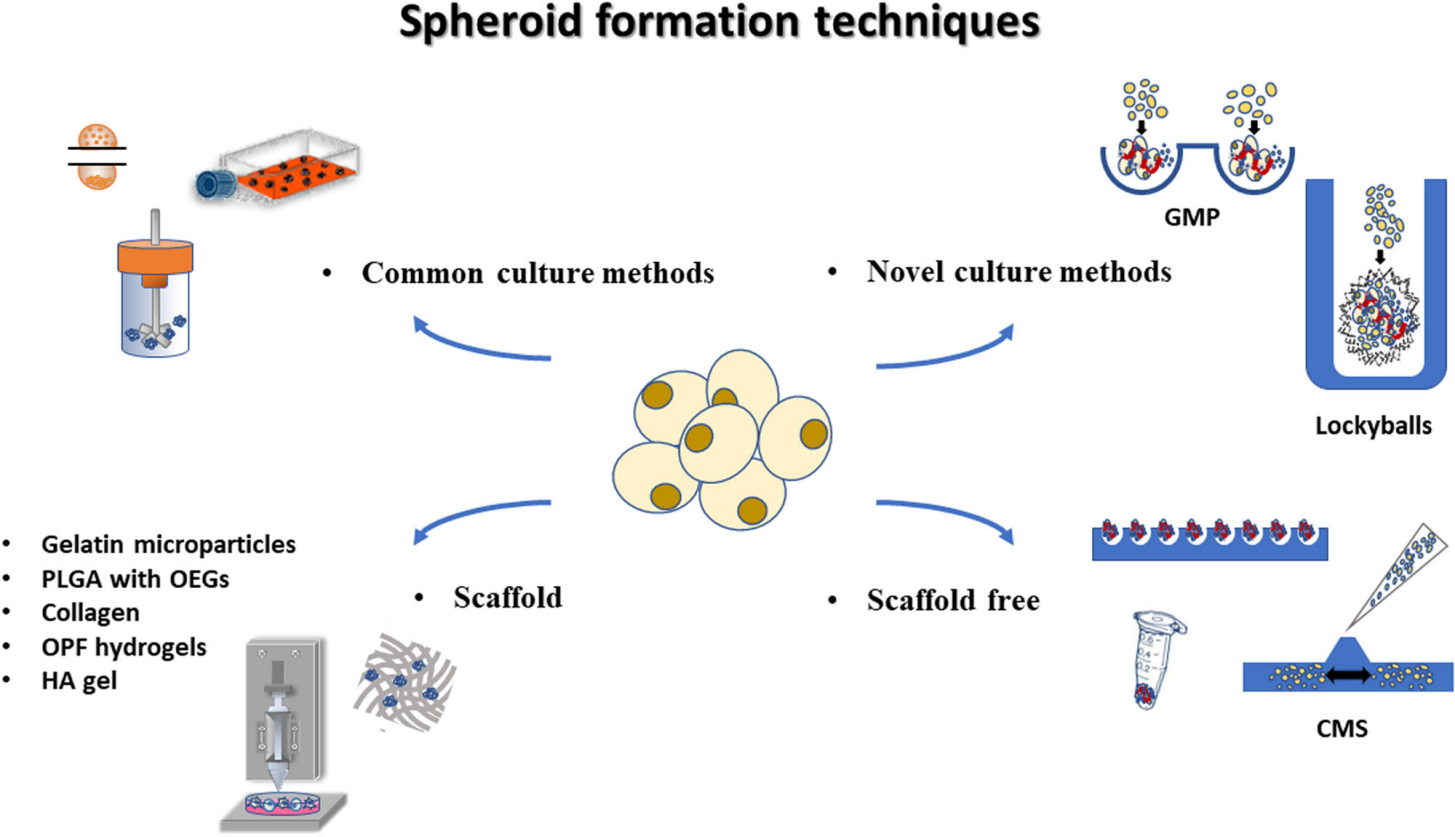
Summary of methods to form spheroids of adipose stem cells. Representative culture methods listed in the text to form spheroids of adipose stem cells. Common culture methods include the use of ultralow adhesion flask, hanging drop and spinner flask. Novel culture methods, for example, include lockyballs (microscaffolds with a porous wall with interlockable hooks) and GMP (microwells plate employed with gelatin microparticles) techniques. Moreover, spheroid formation can take place through scaffold‐free methods (e.g., CMS [centrifugal microfluidic‐based spheroid] or plates treated with Pluronic F‐127 aqueous solution) or by using several materials such as PLGA (Poly [l‐glutamic acid]) with OEGs (Oligo(ethylene glycol), Collagen, OPF (oligo(poly(ethylene glycol) fumarate) hydrogels and HA (hyaluronic acid) gel.

In the near future, clinical applications in regenerative medicine will feature the use of bio‐printers and bioreactors capable of creating *ad personam* organ or tissue replacement. The results of this systematic review suggest that 3D‐ASCs could represent the ideal candidates for clinical studies of regenerative medicine and, given their variety, ad hoc scaffolds could be chosen according to the specific requirements. Figure 1 and 2

## AUTHOR CONTRIBUTIONS

*Conceptualization*: Di Stefano Anna Barbara. *Literature search and data analysis*: Di Stefano Anna Barbara, Urrata Valentina, Trapani Marco *Writing*—*original draft preparation*: Di Stefano Anna Barbara, Urrata Valentina, Trapani Marco; *Writing—review and editing*: Moschella Francesco, Cordova Adriana, and Toia Francesca.

## CONFLICT OF INTEREST

The authors declare no conflict of interest.
